# The Relation Between Cigarette Smoking and Development of Sepsis: A 10-Year Follow-Up Study of Four Million Adults from the National Health Screening Program

**DOI:** 10.1007/s44197-024-00197-6

**Published:** 2024-02-19

**Authors:** Eun Hwa Lee, Kyoung Hwa Lee, Kyu-na Lee, Yebin Park, Kyung Do Han, Sang Hoon Han

**Affiliations:** 1 Divison of Infectious Disease, Department of Internal Medicine, Yonsei University College of Medicine, 211 Eonju-ro, Gangnam-gu, Seoul, 06273 Republic of Korea; 2 Department of Preventive Medicine and Public Health, College of Medicine, Catholic University of Korea, Seoul, Republic of Korea; 3 Department of Statistics and Actuarial Science, Soongsil University, 369 Sangdo-ro, Dongjak-gu, Seoul, 06978 Republic of Korea; 4 Institute for Innovation in Digital Healthcare, Yonsei University College of Medicine, Seoul, Republic of Korea

**Keywords:** Cumulative amount, Sepsis, Cigarette smoking, Incidence, Pack-year

## Abstract

**Background:**

Sepsis remains a growing global health concern with soaring mortality and no direct anti-sepsis drug. Although smoking has distinct deleterious effects on chronic inflammatory illnesses and can impair immune function, a comprehensive analysis of the connection between sepsis and smoking is lacking.

**Methods:**

This large-scale longitudinal cohort study retrospectively assessed adults aged ≥ 20 years who underwent national health checkups under the Korean National Health Insurance Service between January and December 2009 (*N* = 4,234,415) and were followed up for 10 years. Sepsis was identified based on the International Classification of Diseases, 10th Revision codes, and smoking status, including accumulated amount, was collected through a self-administered questionnaire. The Cox proportional hazard regression model was used, adjusting for age, sex, household income, body mass index, drinking, exercise, diabetes, hypertension, dyslipidemia, and chronic renal disease.

**Results:**

After excluding cases with sepsis occurring before follow-up or after ≤ 1 year of follow-up, 3,881,958 participants, including non-smokers (*N* = 2,342,841), former smokers (*N* = 539,850), and active smokers (*N* = 999,267), were included. Compared to non-smokers, all active smokers (adjust hazard ratio: 1.41, 95% confidence interval 1.38–1.44) and former smokers (1.10, 1.07–1.14) with ≥ 20 pack-years exhibited a significantly higher risk of sepsis (*p* < 0.001). Smoking of ≥ 30 pack-years in former and active smokers groups significantly increased sepsis incidence (adjust hazard ratio [95% confidence interval] 1.34 [1.31–1.38], *p* < 0.001).

**Conclusions:**

Smoking is closely associated with the incidence of sepsis. Smoking cessation may help in the primary prevention of sepsis.

**Supplementary Information:**

The online version contains supplementary material available at 10.1007/s44197-024-00197-6.

## Introduction

Sepsis is a life-threatening infection incurred by exaggerated and imbalanced inflammation, constituting a substantially important cause of death and morbidities worldwide [1, 2]. Despite advancements in intensive care, including early recolonization and initial resuscitation, the 30-day mortality attributable to sepsis remains high, exceeding 25–30% [3, 4]. Disturbingly, the high short-term mortality for patients with septic shock, which leads to more detrimental outcomes than sepsis, has not been decreasing recently [3]. The challenges of sepsis management originate from the absence of a specific anti-sepsis drug [5, 6]. Therefore, the current international guideline focuses on preventing or rapid recovery of tissue hypoperfusion-induced end-organ damage [1]. However, the protocol-based bundle approaches could not remarkably improve the prognosis of septic shock, and the most optimal therapeutic targets or goals to fundamentally resolve the intricate pathophysiology of sepsis are still lacking [6–10]. Therefore, prevention of hospital-acquired sepsis and modification of risk factors in lifestyle could be personalized countermeasures to reduce the disease burden of sepsis [11, 12].

Cigarette, a notorious carcinogen with widely documented adverse health impacts, has been recognized as being associated with a range of respiratory and cardiovascular diseases [13–15]. The global population of tobacco users was estimated to reach 1.3 billion in 2020, with smoking-related illnesses claiming approximately 8 million lives in 2019 [16, 17]. Smoking cessation remains a critical action to prevent medical illnesses, disabilities, and fatalities [18].

Many toxic chemicals in cigarettes can impair heterogeneous components of the innate and adaptive immune systems [19–22]. Immune dysfunction and immunosenescence from smoking predispose various organs, including the respiratory tract, to infections caused by pathogens [20, 21, 23–25]. Moreover, smoking-related chronic hyperinflammation and oxidative stress could increase the risk of developing sepsis-associated organ failure and worsen the outcome of sepsis [26–30]. Contrastingly, some studies reported that active smokers had a significantly lower all-cause in-hospital mortality in pneumonia or sepsis compared to that in non-smokers [22, 31–34].

Despite the conflicting findings between smoking and infection severity and the considerable social repercussions of smoking on public health, there are few large-scale evaluations to establish a definitive link between smoking and sepsis incidence. This study aimed to provide potential evidence substantiating the association of smoking with sepsis, utilizing a cohort of large community residents in South Korea, where the adult smoking rate was similar to the average of Organization for Economic Co-operation and Development countries [35].

## Methods

### Study Design and Database Source

This retrospective longitudinal cohort study was based on the healthcare promotion strategy of the Korean National Health Insurance Service (KNHIS), the solitary public insurance system requiring mandatory subscription [36]. The comprehensive system for claims collects medical information encompassing the International Classification of Disease, 10th Revision (ICD-10) diagnostic codes, prescriptions, medical treatments, and procedures in the KNHIS database [36]. The KNHIS provides the health assessment program (HEALS) biennially for individuals aged ≥ 20 years, without additional cost, including anthropometric measurements (height, weight, waist circumference, and blood pressure), a self-administered survey regarding prior/current chronic diseases with medication and health-related behaviors (cigarette smoking, drinking, and exercise), and fasting laboratory tests [37]. The data extraction from the KNHIS–HEALS databases was conducted with anonymized unique identification.

This study was reviewed and approved by the Institutional Review Board of Gangnam Severance Hospital (No. 3-2023-0267) and the National Health Insurance Sharing Service, with a waiver of the requirement for written informed consent. The study was conducted following the principles outlined in the Declaration of Helsinki.

### Cohort Composition

**Fig. 1:**
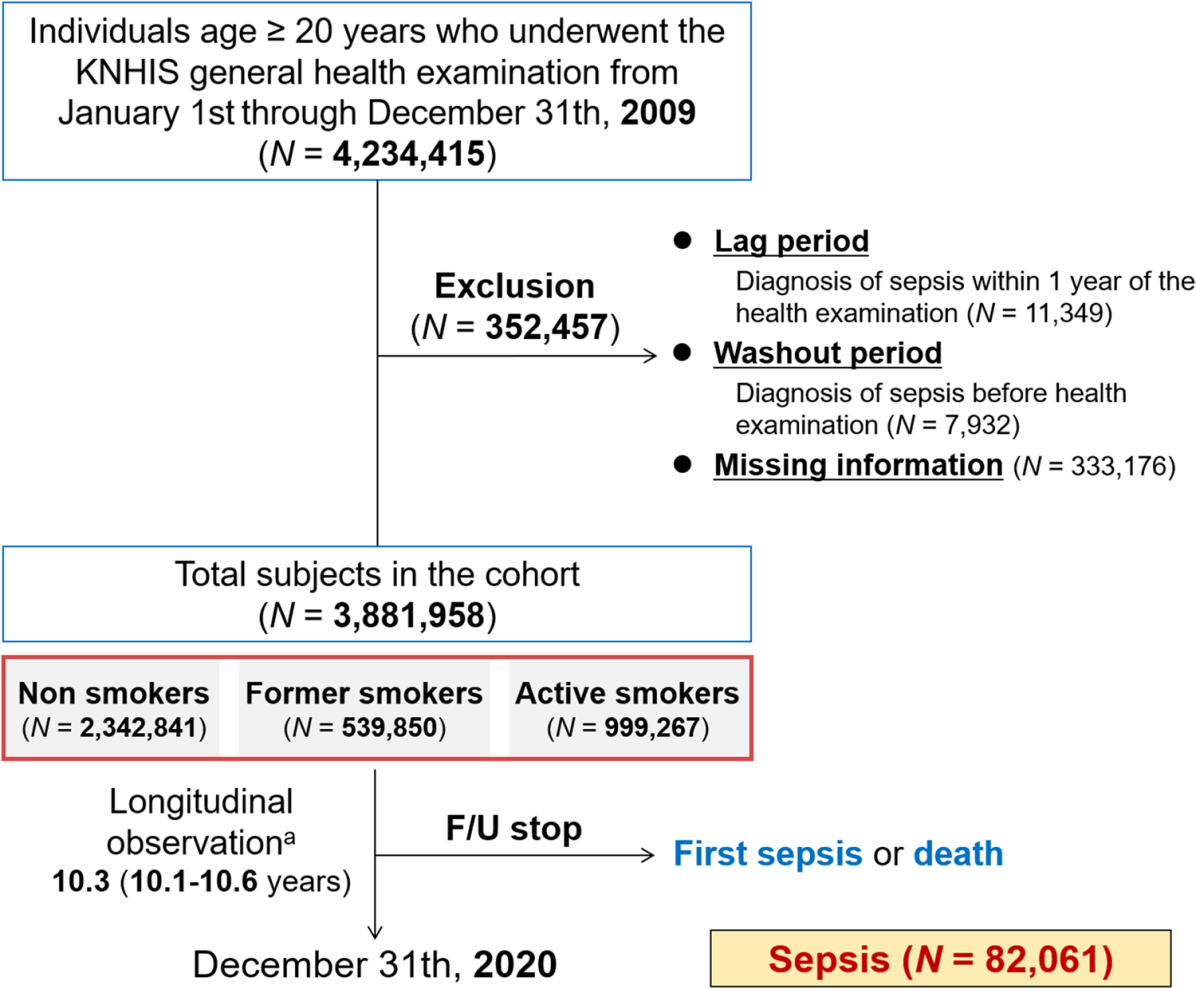
Flow chart for cohort organization and research progress. ^a^Represent median (interquartile range). *F/U* follow-up, *KNHIS* Korean National Health Insurance Service

We screened all examinees (*N* = 4,234,415) in the KNHIS–HEALS databases from January 2009 to December 2009. Time zero was set as the date of health assessment in 2009. Exclusions comprised individuals with missing values (*N* = 333,176) and those previously diagnosed with sepsis during the wash-out period before time zero (*N* = 7,932). I addition, we excluded examinees who developed sepsis within one year from time zero (*N* = 11,349) to evaluate the potential causality between smoking and sepsis. Ultimately, the cohort consisted of 3,881,958 individuals. Follow-up extended from time zero through December 2020, ceasing at the occurrence of the first sepsis or death event; otherwise, it continued (Fig. 1).

### Definitions

Sepsis was identified based on the ICD-10 codes that directly indicate sepsis, similar to those used in other retrospective cohort studies from a large-scale nationwide claims database, except O85 (puerperal sepsis), O88.3 (obstetric pyemic and septic embolism), and P36 (bacterial sepsis of newborn) (Supplementary Table 1) [38]. To further ensure the accuracy of sepsis diagnosis, we considered patients with the ICD-10 codes in the inpatient claims as the sepsis group.

The cigarette smoking behavior was categorized into three groups: non-smokers (participants who have never smoked before time zero), former smokers (participants who smoked but stopped before time zero), and active smokers (participants who continued to smoke at time zero). Total exposure to smoking in former and active smokers was divided into < 10, < 20, < 30, or ≥ 30 pack-years, calculated by multiplying total years by the average daily amount of smoking. The daily number of cigarettes in the self-questionnaire was divided by 20 to convert to pack units (Supplementary Table 2).

The degree of drinking was categorized into abstainers, mild to moderate (< 30 g/day), or heavy drinkers (> 30 g/day) based on the drinking days per week and the amount of alcohol consumed per day [39]. Regular exercise was defined as moderate-intensity physical activities performed ≥ 5 days a week for ≥ 30 min/day or vigorous-intensity physical activities performed ≥ 3 days a week for ≥ 20 min/day [40, 41]. Diabetes mellitus (DM), hypertension, dyslipidemia, and chronic kidney disease were identified based on ICD-10 codes or blood test results in the HEALS applying the diagnostic criteria as of 2009 or medication status at time zero for each disease (Supplementary Table 3). Body mass index (BMI) was subdivided into five levels: underweight (< 18.5 kg/m^2^), normal (18.5–22.9 kg/m^2^), overweight (23–24.9 kg/m^2^), obese I (25–29.9 kg/m^2^), and obese II (≥ 30 kg/m^2^) according to the Asia–Pacific standard of the World Health Organization [42]. The lowest quartile group of income status was defined as an annual household income lower than the 25th percentile based on the 2010 population and Housing Census in South Korea.

### Statistical Analysis

Data were expressed as the median with interquartile range or mean ± standard deviation. The incidence rate (IR) of sepsis was calculated per 1000 person-years. A comparison of three groups according to smoking status was performed using the Kruskal–Wallis test for categorical variables and a one-way analysis of variance test for continuous variables. We evaluated the association between smoking and sepsis using the multivariate Cox proportional hazard regression models, expressing hazard ratio (HR) and 95% confidence interval (CI). Along with the unadjusted analysis (Model 1), the HRs of sepsis incidence were adjusted for age and sex in Model 2, or for age, sex, five levels of BMI, lowest income status, alcohol consumption, regular exercise, DM, hypertension, dyslipidemia, and chronic kidney disease in Model 3. Kaplan–Meier curves were generated to compare the cumulative incidence of sepsis by smoking status or accumulated amount, and significance was assessed using log-rank test. In all analyses using the SAS program (version 9.4), a two-tailed *p* ≤ 0.05 was considered statistically significant.

## Results

### Basic Characteristics

Our cohort was composed of non-smokers (*N* = 2,342,841, 60.4%), former smokers (*N* = 539,850, 13.9%), and active smokers (*N* = 999,267, 25.7%). Sepsis occurred in 82,061 participants during a median follow-up period of 10.3 years. The cumulative amount of cigarette smoking was 10 (5–20) and 12.5 (6.75–20) pack-years in former and active smokers, respectively. The active smokers had the lowest mean age (42.8 ± 12.5 years) and the highest proportion of people aged < 40 years (44.8%) (all *p* < 0.001). The proportion of women among ever-smokers was very low (6%). The former smokers group had the highest level of BMI and frequencies of obesity, DM, hypertension, and dyslipidemia (all *p* < 0.001). In addition to the high rate of metabolic abnormalities, including the highest glucose and total cholesterol values, the participants who exercised regularly were significantly typical in the former smokers compared to that in the non- or active smokers (25.3 vs. 17.0% or 16.3%, *p* < 0.001). The active smokers had a significantly higher frequency of heavy drinking than that in the non-smokers (17.8 vs. 2.4%, *p* < 0.001) (Table 1).

**Table 1:** Baseline characteristic of the cohort population

Characteristics	Total (*N* = 3,881,958)	Smoking status			*p* value
		None (*N* = 2,342,841)	Former (*N* = 539,850)	Active (*N* = 999,267)	
Cumulative amount of smoking, pack-years	11.25 (5.25, 20)	–	10 (5, 20)	12.5 (6.75, 20)	< 0.0001
*Age, years*	47.2 ± 14.0	48.7 ± 14.5	49.1 ± 13.0	42.8 ± 12.5	< 0.0001
< 40	1,200,327 (30.9%)	617,934 (26.4%)	134,512 (24.9%)	447,881 (44.8%)	< 0.0001
40–64	2,173,323 (56.0%)	1,354,507 (57.8%)	332,068 (61.5%)	486,748 (48.7%)	
≥ 65	508,308 (13.1%)	370,400 (15.8%)	73,270 (13.6%)	64,638 (6.5%)	
Sex, male	2,103,636 (54.2%)	651,422 (27.8%)	512,098 (94.9%)	940,116 (94.1%)	< 0.0001
Wait circumference, cm	80.2 ± 9.1	78.3 ± 9.2	84.1 ± 7.8	82.6 ± 8.3	< 0.0001
*BMI, kg/m*^*2*^	23.7 ± 3.2	23.5 ± 3.3	24.3 ± 2.9	23.9 ± 3.3	< 0.0001
< 18.5	143,796 (3.7%)	102,229 (4.4%)	9,281 (1.7%)	32,286 (3.2%)	< 0.0001
< 23	1,516,946 (39.1%)	982,802 (42.0%)	163,041 (30.2%)	371,103 (37.1%)	
< 25	955,570 (24.6%)	553,756 (23.6%)	153,714 (28.5%)	248,100 (24.8%)	
< 30	1,127,768 (29.1%)	624,114 (26.6%)	195,850 (36.3%)	307,804 (30.8%)	
≥ 30	137,878 (3.6%)	79,940 (3.4%)	17,964 (3.3%)	39,974 (4.0%)	
*Underlying diseases*					
Diabetes mellitus	335,451 (8.6%)	188,465 (8.0%)	60,298 (11.2%)	86,688 (8.7%)	< 0.0001
Hypertension	986,386 (25.4%)	601,608 (25.7%)	169,129 (31.3%)	215,649 (21.6%)	< 0.0001
Dyslipidemia	672,774 (17.3%)	422,240 (18.0%)	102,862 (19.1%)	147,672 (14.8%)	< 0.0001
Chronic kidney disease	268,912 (6.9%)	178,286 (7.6%)	40,954 (7.6%)	49,672 (5.0%)	< 0.0001
*Income status*					< 0.0001
Lowest quartile	758,285 (19.5%)	519,410 (22.2%)	74,399 (13.8%)	164,476 (16.5%)	
*Alcohol drinking*					< 0.0001
None	2,013,686 (51.9%)	1,619,562 (69.1%)	161,378 (29.9%)	232,746 (23.3%)	
Mild to moderate	1,561,781 (40.2%)	666,651 (28.5%)	306,828 (56.8%)	588,302 (58.9%)	
Heavy	306,491 (8.0%)	56,628 (2.4%)	71,644 (13.3%)	178,219 (17.8%)	
Regular exercise	696,322 (17.9%)	397,373 (17.0%)	136,597 (25.3%)	162,352 (16.3%)	< 0.0001
*Laboratory findings*					
Fasting glucose, mg/dL	97.3 ± 23.8	96.2 ± 22.2	100.4 ± 25.1	98.1 ± 26.3	< 0.0001
Total cholesterol, mg/dL	195.1 ± 36.8	195.0 ± 37.0	196.0 ± 36.3	194.6 ± 36.6	< 0.0001
LDL cholesterol, mg/dL	113.6 ± 38.6	114.9 ± 38.9	113.8 ± 37.5	110.4 ± 38.3	< 0.0001
HDL cholesterol, mg/dL	56.1 ± 27.9	57.9 ± 30.0	53.7 ± 24.6	53.2 ± 23.8	< 0.0001

Data are expressed as number (percentage) or mean ± standard deviation or median (interquartile range)

*BMI* body mass index, *HDL* high-density lipoprotein, *LDL* low-density lipoprotein, *Q* quartile

### Comparison of Sepsis Incidence in Different Smoking Status and Amount

We observed a progressive increase in the IRs of sepsis with the more cumulative amount of smoking, ranging from 1.25 (< 10 pack-years) to 4.08 (≥ 20 pack-years) in former smokers and from 0.86 (< 10 pack-years) to 3.26/1000 person-years (≥ 20 pack-years) in active smokers. Total active smokers had a significantly higher risk of sepsis in model 2 than that in non-smokers, who were used as the reference (adjusted HR [aHR]: 1.41, 95% CI 1.38–1.44, and *p* < 0.001) (Table 2). The risk of sepsis in active smokers increased with a higher cumulative amount (aHR = 1.39 and 1.42 in < 10 and ≥ 20 pack-years, respectively) (Table 2). However, the risk of sepsis was significantly higher in former smokers than in non-smokers only if they had smoked ≥ 20 packs in total (aHR [95% CI] 1.07 [1.04–1.10]) or for ≥ 20 years (1.07 [1.04–1.09]), or had a cumulative amount of ≥ 20 pack-years (1.10 [1.07–1.14]) (Table 2 and Supplementary Table 4 and 5).

**Table 2:** Impact of smoking status and/or accumulated amount on incidence of sepsis

Smoking	Subjects	Sepsis	Duration (years)	IR^a^	HR (95% CI)		
					Model 1	Model 2	Model 3
None	2,342,841	51,407	23,688,509.1	2.17	1 (Refence)	1 (Refence)	1 (Refence)
*Former*	539,850	12,247	5,408,997.5	2.26	1.049 (1.029, 1.070)	0.993 (0.971, 1.016)	1.006 (0.983, 1.030)
< 10 pack-years	229,757	2,910	2,335,973.1	1.25	0.575 (0.554, 0.597)	0.876 (0.842, 0.911)	0.904 (0.869, 0.941)
< 20 pack-years	146,770	2,841	1,479,227.1	1.92	0.889 (0.856, 0.923)	0.910 (0.875, 0.947)	0.933 (0.897, 0.971)
≥ 20 pack-years	163,323	6,496	1,593,797.3	4.08	1.901 (1.852, 1.949)	1.108 (1.077, 1.140)	1.103 (1.072, 1.136)
*p* value					< 0.0001	< 0.0001	< 0.0001
*Active*	999,267	18,407	10,030,402.3	1.84	0.852 (0.838, 0.867)	1.427 (1.398, 1.457)	1.407 (1.378, 1.437)
< 10 pack-years	357,115	3,144	3,639,611.2	0.86	0.402 (0.386, 0.414)	1.385 (1.334, 1.438)	1.385 (1.334, 1.439)
< 20 pack-years	300,248	4,301	3,032,767.8	1.42	0.658 (0.637, 0.678)	1.390 (1.344, 1.437)	1.388 (1.342, 1.435)
≥ 20 pack-years	341,904	10,962	3,358,023.4	3.26	1.523 (1.492, 1.555)	1.455 (1.421, 1.491)	1.422 (1.387, 1.457)
*p* value					< 0.0001	< 0.0001	< 0.0001
*p* value^b^					< 0.0001	< 0.0001	< 0.0001

Model 1: Non-adjusted, Model 2: Adjusted with age and sex, Model 3: Adjusted with age, sex, lowest income status, five categories of body-mass index, regular exercise, alcohol consumption, diabetes mellitus, hypertension, dyslipidemia, chronic kidney disease

*Ex* ex-smoker, *CI* confidence interval, *HR* hazard ratio, *IR* incidence rate

^a^Per 1000 person-years

^b^Comparison between non-smoking, ex-smoking, and current smoking

**Fig. 2:**
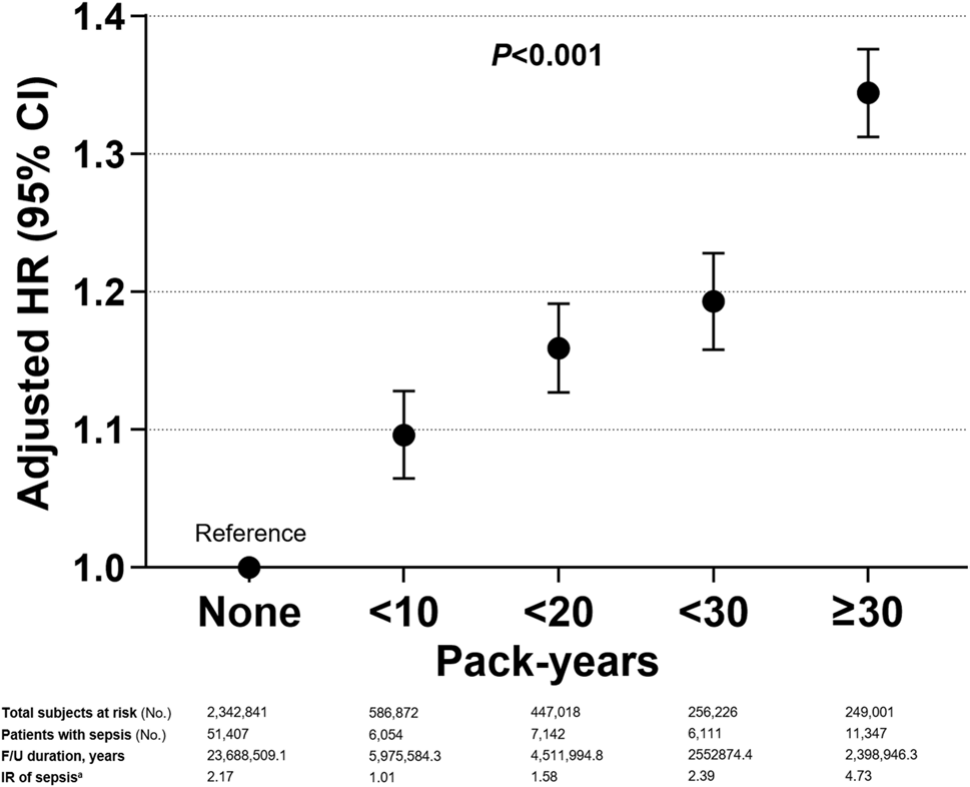
Effect of lifetime cumulative smoking amount on sepsis incidence regardless of smoking status at the time of health examination. The circles represent the hazard ratio adjusted with age, sex, lowest income status, five categories of body-mass index, regular exercise, alcohol consumption, diabetes mellitus, hypertension, dyslipidemia, chronic kidney disease, and the top and bottom of the bar represent the upper and lower values of the 95% confidence interval, respectively. ^a^Per 1,000 person-years. *F/U* follow-up, *CI* confidence interval, *HR* hazard ratio, *IR* incidence rate, *No* number

Analysis of total cumulative amount of smoking until time zero without considering smoking status at the time of starting follow-up showed that ever-smokers exhibited elevated risk of sepsis with greater levels of smoking exposure (aHR [95% CI] in model 2, 1.10 [1.07–1.13] in < 10 pack-years, 1.16 [1.13–1.19] in 10–20 pack-years, 1.19 [1.16–1.23] in 20–30 pack-years, and 1.34 [1.31–1.38] in ≥ 30 pack-years, *p* < 0.001) (Fig. 2 and Supplementary Table 6).

**Fig. 3:**
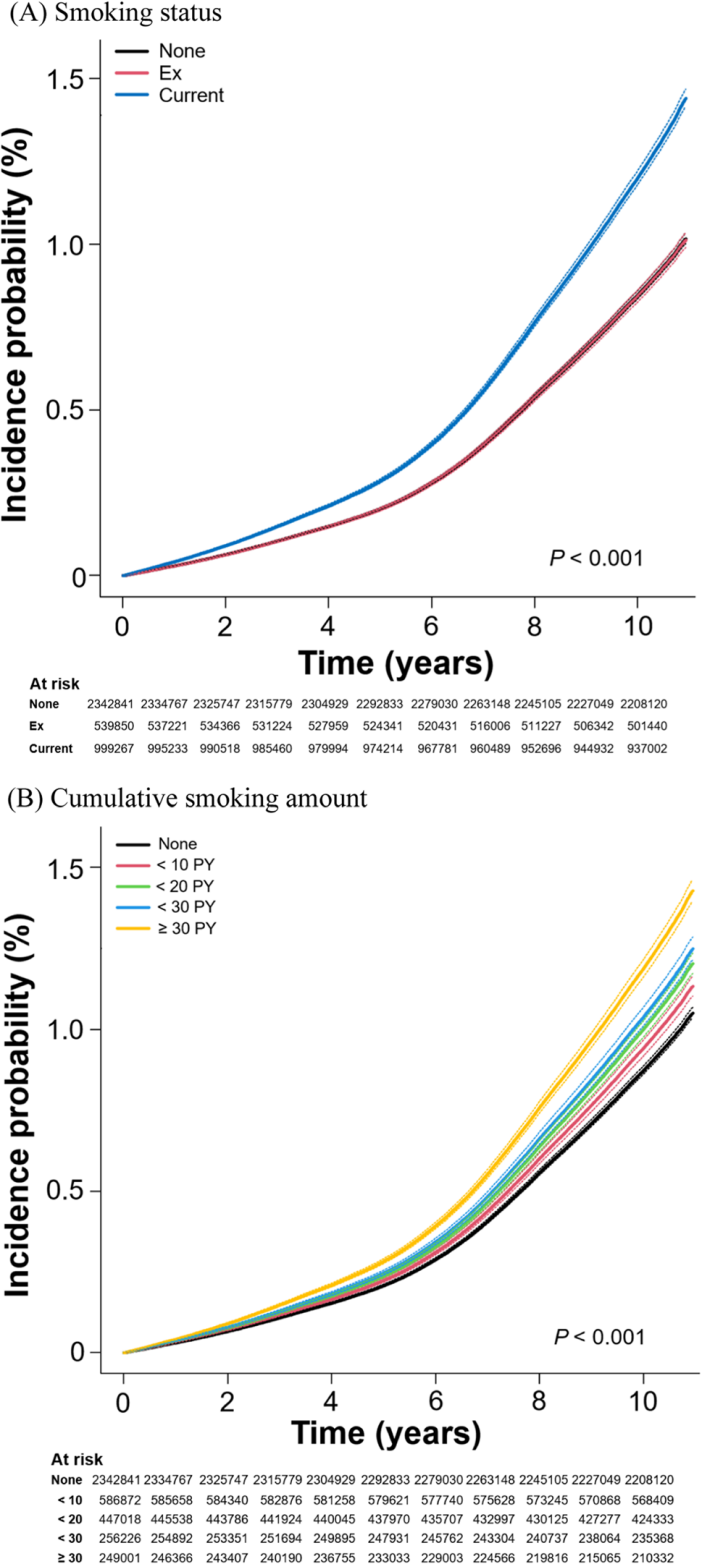
Incidence probability of sepsis incidence according to smoking status or total accumulated amount during the entire observation period. **A** Smoking status. **B** Cumulative smoking amount. All incidence probabilities were adjusted by with age, sex, lowest income status, five categories of body-mass index, regular exercise, alcohol consumption, diabetes mellitus, hypertension, dyslipidemia, chronic kidney disease. *Ex* ex-smoker, *PY* pack-years

The Kaplan–Meier curve also showed that the incidence of sepsis at time zero was significantly higher in active smokers than in non-smokers or former smokers, with a larger difference over time (*p* < 0.001). The incidence probability of sepsis was consistent in the non-smoker and former–smoker groups throughout the follow-up period. In addition, the occurrence of sepsis increased gradationally with the higher cumulative amount regardless of smoking status at the time of examination (*p* < 0.001) (Fig. 3).

### Risk of Sepsis According to Smoking Amount and Various Variables

The effect of smoking on the incidence of sepsis in women was higher than that in men (aHR [95% CI] in 0–10 pack-years from model 2, 1.50 [1.41–1.60] vs. 1.02 [0.98–1.05], *p* < 0.001). In the group aged ≥ 65 years with the highest IR of sepsis among all subgroups, smoking was significantly associated with an increased risk of sepsis (aHR [95% CI] in former and active smokers, 1.08 [1.05–1.12] and 1.45 [1.41–1.49], respectively, *p* < 0.001). However, smoking amount or status in participants aged < 40 years did not increase the risk of sepsis. Non-obese participants with a BMI of < 25 kg/m^2^ had a significantly higher incidence of sepsis than that observed in the obese participants at the same amount of smoking (Supplementary Fig. 1 and Table 7).

Even in groups that exercised regularly and abstained from alcohol as good health habits, smoking increased the incidence of sepsis (Supplementary Fig. 2 and Table 7). Smokers without DM (*p* for interaction < 0.001) or hypertension (*p* for interaction = 0.044) were significantly associated with a higher risk of sepsis than those without DM or hypertension, respectively (Supplementary Fig. 3 and Table 7).

## Discussion

Our results provide evidence that cigarette smoking is a valid risk factor contributing to sepsis occurrence. Data from models adjusting for sepsis risk factors and subgroup analyses of various variables sufficiently support this conclusion. The association between smoking and increased incidence of sepsis, regardless of chronic illnesses or health behaviors (especially in cases of heavy smoking), suggests that smoking may independently affect sepsis development.

A major findings was that the positive relation between the accumulated amount of smoking and a higher risk of sepsis, even in former smokers. Active smokers with prolonged smoking exhibited a higher hazard than former or non-smokers. The incidence of sepsis was particularly increased among those who smoked ≥ 30 pack-years. However, former smokers who quit smoking with a small amount and/or a short duration had about a 10% lower risk of sepsis compared to non-smokers (aHR [95% CI] 0.90 [0.87–0.94] in < 10 pack-years and 0.93 [0.90–0.97] in 10–20 pack-years) (Table 2 and Supplementary Table 5 and 6). The influence of smoking was evident in older people aged ≥ 65 years, where the risk and poor outcome of sepsis substantially increased. The potential for smoking cessation to prevent critical infections need to be proven through interventional approaches, along with efforts to encourage smoking cessation.

Previous immunologic experiments reported that smoking has been associated with impaired phagocytic function and cytokine expression of T lymphocyte and macrophage in the reparatory tract or peripheral blood [19–22, 26, 43–46]. These studies collectively underscore the detrimental impact of smoking on the immune system, which persists even after smoking cessation. In addition, Huttunen et al. highlighted smoking as an independent risk factor for case fatality among 149 patients with bacteremia, with active smokers requiring more frequent intensive care unit care and mechanical ventilation compared to that required by non-smokers [47]. Moreover, the longitudinal cohort of the American National Center for Health Statistics showed that smokers had a larger hazard of death caused by septicemia (A40–A41 of the ICD-10 codes), which was approximately twice as high as those of non-smokers [30]. The report by Kempker et al. [30] aligned with our results, focusing on the risk of developing sepsis from a larger number of patients, including septic shock, and more detailed analyses about smoking duration and cumulative smoking amount.

Strategies to prevent prejudicial outcomes from sepsis include minimizing healthcare facility-associated infections and controlling progression to multi-organ dysfunctions after sepsis [11]. Conventional measures against infection, such as improved sanitation and enhanced herd immunity through vaccination, have been enormously effective as primary prevention for sepsis. However, public efforts to decrease the disease burden of sepsis by managing modifiable risk factors in the population have not received adequate attention. As data accumulates on the relation between smoking and sepsis, the alteration of smoking behavior as a lifestyle habit that aggravates many diseases will be quite cost-effective by blocking the occurrence of sepsis itself from the public health perspective. The negative impact of smoking on severe infection through immune dysfunction may also need to be highlighted to the same extent as in malignancies and cardiovascular diseases.

This study has several limitations. First, extracting sepsis events with diagnostic difficulties from the large database using ICD-10 codes may have resulted in a loss of cases or inclusion imprecision. A large number of events must be included to secure sufficient statistical power; however, prospectively or retrospectively defining sepsis through extensive review by medical staff would make big data analysis virtually unfeasible. Even though the incidence of sepsis can be affected by various factors, sepsis IR in our cohort (211/100,000 person-years) was within the same range as the Korean population-based study (266–453/100,000 person-years) and the meta-analysis of international epidemiology (148–288/100,000 person-years) [48–50]. This suggests that our sepsis screening did not deviate significantly from the general assessment. Second, smoking status and amount surveyed through self-questionnaires might not exactly match reality. In addition, the smoking history assessed once at time zero may have changed during the 10-year follow-up. The smoking rate among Korean adult men has steadily decreased from 47.0% in 2009 to 31.3% in 2021 [35]. Therefore, a substantial number of participants among active smokers at health examination may have stopped smoking during follow-up. However, among self-described lifestyle habits, smoking behavior is judged to be the item with a lower probability of being falsely stated. Moreover, dividing the total smoking amount into 10-pack-year intervals could reduce bias due to smoking cessation during follow-up. Despite the possibility of changes in smoking status, our data may have meaningful implications that the cumulative amount of smoking before a certain point can predict an increased risk of sepsis. Third, in line with the characteristics of the Korean population, with the lowest female smoking rate among Organization for Economic Co-operation and Development member nations [35], almost all former or active smokers in our cohort were men. Last, we investigated only cigarettes among various vaporizing tobacco products.

## Conclusions

This large longitudinal cohort study presents that cigarette smoking status and cumulative smoking amounts are independently associated with a higher incidence of sepsis. The risk of sepsis was higher even for heavy former smokers than for non-smokers. The older smoking population, with basically the highest incidence and worst outcomes of sepsis due to various risk factors, was especially vulnerable to sepsis. With the increasing awareness that sepsis is a public health problem that can be prevented by smoking cessation, these findings suggest that sepsis also needs to be highlighted in public notice and prevention campaigns regarding the adverse health effects of smoking.

### Supplementary Information

Below is the link to the electronic supplementary material.

Supplementary file1 (DOCX 677 KB)

## Data Availability

Not applicable.
