# Supplementary material for: The Relation Between Cigarette Smoking and Development of Sepsis: A 10-Year Follow-Up Study of Four Million Adults from the National Health Screening Program
Source: J Epidemiol Glob Health. 2024 Feb 19;14(2):444–52. doi: 10.1007/s44197-024-00197-6 (PMC11176127; doi:10.1007/s44197-024-00197-6)
Supplement: Supplementary file 1 — Supplementary file1 (DOCX 677 KB) [file 44197_2024_197_MOESM1_ESM.docx]

**Supplemental Online Content**

KH Lee, EH Lee, K Lee, et al. The Relation between Cigarette Smoking and Development of Sepsis: A 10-year Follow-up Study of Four Million Adults from the National Health Screening Program

**Supplementary Table 1**. List of diagnosis codes used to extract patients with sepsis

**Supplementary Table 2.** Self-report questionnaires from the biennial health screening program by the Korean National Health Insurance Service

**Supplementary Table 3.** List of diagnosis codes and criteria to identify chronic medical illnesses

**Supplementary Table 4**. Impact of smoking status and amount on incidence of sepsis

**Supplementary Table 5.** Impact of smoking status and duration on incidence of sepsis

**Supplementary Table 6**. Impact of total cumulative smoking amount regardless of smoking status on health examination on incidence of sepsis

**Supplementary Table 7**. Impact of smoking status or amount on incidence of sepsis according to various characteristics

**Supplementary Fig. 1**. Impact of total smoking amount on incidence of sepsis by baseline characteristics

**Supplementary Fig. 2**. Impact of total smoking amount on incidence of sepsis by lifestyle behaviors

**Supplementary Fig. 3**. Impact of total smoking amount on incidence of sepsis by medical comorbidities

**Supplementary Table 1.** List of diagnosis codes used to extract patients with sepsis

| **ICD-10 code** | **Diagnosis** |
| --- | --- |
| A02.1 | Salmonella sepsis |
| A20.7 | Septicemic plague |
| A22.7 | Anthrax sepsis |
| A26.7 | Erysipelothrix sepsis |
| A32.7 | Listerial sepsis |
| **A40** | ***Streptococcal* sepsis** |
| A40.0 | Sepsis due to *Streptococcus*, group A |
| A40.1 | Sepsis due to *Streptococcus*, group B |
| A40.2 | Sepsis due to *Streptococcus*, group D and *enterococcus* |
| A40.3 | Sepsis due to *Streptococcus pneumoniae* (Pneumococcal sepsis) |
| A40.8 | Other *Streptococcal* sepsis |
| A40.9 | *Streptococcal* sepsis, unspecified |
| **A41** | **Other sepsis** |
| A41.0 | Sepsis due to *Staphylococcus aureus* |
| A41.1 | Sepsis due to other specified *Staphylococcus*  (Sepsis due to coagulase-negative *Staphylococcus*) |
| A41.2 | Sepsis due to unspecified *Staphylococcus* |
| A41.3 | Sepsis due to *Haemophilus influenzae* |
| A41.4 | Sepsis due to anaerobes |
| A41.5 | Sepsis due to other Gram-negative organisms  (Gram-negative sepsis NOS) |
| A41.8 | Other specified sepsis |
| A41.9 | Sepsis, unspecified (Septicemia) |
| A42.7 | Actinomycotic sepsis |
| B37.7 | Candidal sepsis |
| R57.2 | Septic shock |
| **R65** | **Systemic Inflammatory Response Syndrome** |
| R65.0 | Systemic Inflammatory Response Syndrome of infectious origin without organ failure |
| R65.1 | Systemic Inflammatory Response Syndrome of infectious origin with organ failure (Severe sepsis) |

No patients were defined by ICD-10 codes A20.7 and A22.7.

Abbreviations: ICD-10, International Classification of Diseases and Related Health Problems, 10th Revision; NOS, not otherwise specified.

**Supplementary Table 2.** Self-report questionnaires from the biennial health screening program by the Korean National Health Insurance Service

| **Physical activities**  Please read the following items and mark '√' on the response that corresponds to your activity status during the past week. | | | | | | | | | |
| --- | --- | --- | --- | --- | --- | --- | --- | --- | --- |
| **1**. How many days in the past week did you engage in intense activities that made you breathe much harder than usual for more than 20 minutes? (e.g., running, aerobics, fast cycling, hiking, etc.) | | | | | | | | | |
| 0 | 1 | 2 | | 3 | 4 | 5 | | 6 | 7 |
| **2**. How many days in the past week did you engage in moderate activities that made you breathe slightly harder than usual for more than 30 minutes? (e.g., brisk walking, playing tennis, cycling at a regular pace, mopping, etc.) Exclude activities related to the response in 1. | | | | | | | | | |
| 0 | 1 | 2 | | 3 | 4 | 5 | | 6 | 7 |
| **3**. How many days in the past week did you walk for at least 30 minutes, combining walks of at least 10 minutes each time? (e.g., walking during commuting or leisure time) Exclude physical activities related to the responses in 1 and 2. | | | | | | | | | |
| 0 | 1 | 2 | | 3 | 4 | 5 | | 6 | 7 |
| **Smoking habits** | | | | | | | | | |
| Please read the following items and provide information that corresponds to your current condition. | | | | | | | | | |
| **1**. Have you smoked a total of 5 packs (100 cigarettes) or more in your lifetime? | | | | | | | | | |
| (1) No | | | (2) Yes, but I quit.  → Go to questionnaire 2 | | | | (3) Yes, I am smoking.  → Go to questionnaire 3 | | |
| **2**. If you are an ex-smoker, | | | | | | | | | |
| \| How many years did you smoke? \| Total ______ years \| \| --- \| --- \| \| How many cigarettes per day? \| ______ cigarettes \| | | | | | | | | | |
| **3**. If you are a current smoker, | | | | | | | | | |
| \| How many years have you been smoking? \| Total ______ years \| \| --- \| --- \| \| How many cigarettes per day? \| ______ cigarettes \| | | | | | | | | | |
| **Drinking habits** | | | | | | | | | |
| Please read the following items and provide information that corresponds to your current condition. | | | | | | | | | |
| **1**. On average, how many days a week do you drink alcohol? | | | | | | | | | |
| 0 | 1 | 2 | | 3 | 4 | 5 | | 6 | 7 |
| **2**. How much do you usually drink per day (regardless of the type of alcohol)? | | | | | | | | | |
| ______ glasses | | | | | | | | | |

**Supplementary Table 3.** List of diagnosis codes and criteria to identify chronic medical illnesses

| **ICD-10 code** | **ICD-10 Code** | **Diagnostic criteria** |
| --- | --- | --- |
| **DM** | **E10** - Type 1 DM | Fasting glucose ≥ 126 mg/dL |
|  | **E11** - Type 2 DM |  |
|  | **E11** - Malnutrition-related DM |  |
|  | **E13** - Other specified DM |  |
|  | **E14** - Unspecified DM |  |
| **Hypertension** | **I10** - Essential (primary) hypertension | Systolic BP ≥ 140 mmHg or diastolic BP ≥ 90 mmHg |
|  | **I11** - Hypertensive heart disease |  |
|  | **I12** - Hypertensive renal disease |  |
|  | **I13** - Hypertensive heart and renal disease |  |
|  | **I15** - Secondary hypertension |  |
| **Dyslipidemia** | **E78.0** - Pure hypercholesterolaemia | Fasting total cholesterol ≥ 240 mg/dL |
|  | **E78.1** - Pure hyperglyceridaemia |  |
|  | **E78.2** - Mixed hyperlipidaemia |  |
|  | **E78.4** - Other hyperlipidaemia |  |
|  | **E78.5** - Hyperlipidaemia, unspecified |  |
| **CKD** | **N18.3** - Chronic kidney disease, stage 3, GFR (30-59 mL/min) | Estimated GFR < 60 mL/min/1.73m^2^, as calculated by the Modification of Diet in Renal Disease equation |
|  | **N18.4** - Chronic kidney disease, stage 4, GFR (15-29 mL/min) |  |
|  | **N18.5** - Chronic kidney disease, stage 5, End stage kidney disease |  |
|  | **N18.9** - Chronic kidney disease, unspecified |  |

Abbreviations: BP, blood pressure; CKD, chronic kidney disease; DM, diabetes mellitus; GFR, glomerular filtration rate; ICD-10, International Classification of Diseases and Related Health Problems, 10th Revision.

**Supplementary Table 4**. Impact of smoking status and amount on incidence of sepsis

| **Smoking status** | **Subjects** | **Sepsis** | **Duration** **(years)** | **IR**^a^ | **HR (95% CI)** | | |
| --- | --- | --- | --- | --- | --- | --- | --- |
|  |  |  |  |  | **Model 1** | **Model 2** | **Model 3** |
| **None** | 2,342,841 | 51,407 | 23,688,509.1 | 2.17 | 1 (Refence) | 1 (Refence) | 1 (Refence) |
| **Former smoking &**  **< 10 packs** | 77,248 | 1,482 | 778,123.0 | 1.90 | 0.882 (0.837, 0.929) | 0.952 (0.903, 1.003) | 0.980 (0.930, 1.033) |
| **Former smoking &**  **< 20 packs** | 214,398 | 3,908 | 2,162,983.1 | 1.81 | 0.836 (0.809, 0.864) | 0.901 (0.870, 0.932) | 0.927 (0.895, 0.960) |
| **Former smoking &**  **≥ 20 packs** | 248,204 | 6,857 | 2,467,891.4 | 2.78 | 1.291 (1.258, 1.324) | 1.071 (1.041, 1.101) | 1.067 (1.038, 1.098) |
| **Active smoking &**  **< 10 packs** | 109,277 | 2,465 | 1,087,617.4 | 2.27 | 1.054 (1.012, 1.098) | 1.382 (1.326, 1.440) | 1.396 (1.339, 1.455) |
| **Active smoking &**  **< 20 packs** | 425,980 | 6,646 | 4,296,484.9 | 1.55 | 0.718 (0.698, 0.736) | 1.364 (1.326, 1.402) | 1.363 (1.325, 1.402) |
| **Active smoking &**  **≥ 20 packs** | 464,010 | 9,296 | 4,646,300.1 | 2.00 | 0.929 (0.911, 0.951) | 1.498 (1.461, 1.537) | 1.448 (1.411, 1.486) |
| ***p* value** |  |  |  |  | **< 0.001** | **< 0.001** | **< 0.001** |

Model 1: Non-adjusted, Model 2: Adjusted with age and sex, Model 3: Adjusted with age, sex, income status of the lowest quartile, five categories of body-mass index, regular exercise, alcohol consumption, diabetes mellitus, hypertension, and dyslipidemia, chronic kidney disease. ^a^ Per 1,000 person-years.

Aberrations: CI, confidence interval; HR, hazard ratio; IR, incidence rate.

**Supplementary Table 5.** Impact of smoking status and duration on incidence of sepsis

| **Smoking status** | **Subjects** | **Sepsis** | **Duration** **(Years)** | **IR**^a^ | **HR (95% CI)** | | |
| --- | --- | --- | --- | --- | --- | --- | --- |
|  |  |  |  |  | **Model 1** | **Model 2** | **Model 3** |
| **None** | 2,342,841 | 51,407 | 23,688,509.1 | 2.17 | 1 (Refence) | 1 (Refence) | 1 (Refence) |
| **Former smoking &**  **< 10 years** | 125,089 | 1,258 | 1,275,870.3 | 0.99 | 0.455 (0.431, 0.481) | 0.892 (0.842, 0.944) | 0.916 (0.865, 0.969) |
| **Former smoking &**  **< 20 years** | 190,279 | 2,431 | 1,934,917.5 | 1.26 | 0.580 (0.557, 0.604) | 0.858 (0.822, 0.895) | 0.879 (0.844, 0.919) |
| **Former smoking &**  **≥ 20 years** | 224,482 | 8,558 | 2,198,209.7 | 3.89 | 1.813 (1.772, 1.855) | 1.058 (1.031, 1.086) | 1.065 (1.038, 1.093) |
| **Active smoking &**  **< 10 years** | 143,306 | 972 | 1,464,704.3 | 0.66 | 0.307 (0.288, 0.327) | 1.492 (1.396, 1.589) | 1.473 (1.380, 1.571) |
| **Active smoking &**  **< 20 years** | 372,622 | 2,548 | 3,807,177.2 | 0.67 | 0.310 (0.298, 0.323) | 1.442 (1.382, 1.505) | 1.421 (1.361, 1.483) |
| **Active smoking &**  **≥ 20 years** | 483,339 | 14,887 | 4,758,520.8 | 3.13 | 1.458 (1.432, 1.485) | 1.419 (1.389, 1.451) | 1.399 (1.368, 1.430) |
| ***p* value** |  |  |  |  | **< 0.001** | **< 0.001** | **< 0.001** |

Model 1: Non-adjusted, Model 2: Adjusted with age and sex, Model 3: Adjusted with age, sex, income status of the lowest quartile, five categories of body-mass index, regular exercise, alcohol consumption, diabetes mellitus, hypertension, and dyslipidemia, chronic kidney disease. ^a^ Per 1,000 person-years.

Aberrations: CI, confidence interval; HR, hazard ratio; IR, incidence rate.

**Supplementary Table 6**. Impact of total cumulative smoking amount regardless of smoking status on health examination on incidence of sepsis

| **Pack-years** | **Subjects** | **Sepsis** | **Duration** **(Years)** | **IR**^a^ | **HR (95% CI)** | | |
| --- | --- | --- | --- | --- | --- | --- | --- |
|  |  |  |  |  | **Model 1** | **Model 2** | **Model 3** |
| **None** | 2,342,841 | 51,407 | 23,688,509.1 | 2.17 | 1 (Refence) | 1 (Refence) | 1 (Refence) |
| **< 10** | 586,872 | 6,054 | 5,975,584.3 | 1.01 | 0.468 (0.456, 0.481) | 1.078 (1.048, 1.110) | 1.096 (1.065, 1.128) |
| **< 20** | 447,018 | 7,142 | 4,511,994.8 | 1.58 | 0.733 (0.716, 0.752) | 1.148 (1.117, 1.181) | 1.159 (1.127, 1.191) |
| **< 30** | 256,226 | 6,111 | 2,552,874.4 | 2.39 | 1.113 (1.083, 1.142) | 1.199 (1.164, 1.234) | 1.193 (1.158, 1.228) |
| **≥ 30** | 249,001 | 11,347 | 2,398,946.3 | 4.73 | 2.218 (2.173, 2.263) | 1.383 (1.351, 1.416) | 1.344 (1.312, 1.376) |
| ***p* value** |  |  |  |  | **<0.001** | **<0.001** | **<0.001** |

Model 1: Non-adjusted, Model 2: Adjusted with age and sex, Model 3: Adjusted with age, sex, income status of the lowest quartile, five categories of body-mass index, regular exercise, alcohol consumption, diabetes mellitus, hypertension, dyslipidemia, and chronic kidney disease. ^a^ Per 1,000 person-years.

Aberrations: CI, confidence interval; HR, hazard ratio; IR, incidence rate.

**Supplementary Table 7**. Impact of smoking status or amount on incidence of sepsis according to various characteristics

| **Subgroup** | | **Smoking status** | **Subject** | **Sepsis** | **IR**^a^ | **HR (95% CI)** | | |
| --- | --- | --- | --- | --- | --- | --- | --- | --- |
|  |  |  |  |  |  | **Model 1** | **Model 2** | **Model 3** |
| **Sex** | **Male** | **None** | 651,422 | 15,958 | 2.45 | 1 (Ref.) | 1 (Ref.) | 1 (Ref.) |
|  |  | **Former** | 512,098 | 11,777 | 2.30 | 0.937 (0.915, 0.960) | 0.966 (0.944, 0.990) | 0.980 (0.956, 1.003) |
|  |  | **Active** | 940,116 | 16,924 | 1.79 | 0.734 (0.718, 0.750) | 1.384 (1.354, 1.415) | 1.363 (1.333, 1.394) |
|  | **Female** | **None** | 1,691,419 | 35,449 | 2.06 | 1 (Ref.) | 1 (Ref.) | 1 (Ref.) |
|  |  | **Former** | 27,752 | 470 | 1.68 | 0.818 (0.747, 0.896) | 1.275 (1.164, 1.396) | 1.263 (1.153, 1.384) |
|  |  | **Active** | 59,151 | 1,483 | 2.50 | 1.220 (1.158, 1.285) | 1.654 (1.571, 1.743) | 1.644 (1.561, 1.732) |
|  | *p* for interaction | |  |  |  | <.0001 | <.0001 | <.0001 |
| **Age group,**  **years** | **< 40** | **None** | 617,934 | 2,056 | 0.32 | 1 (Ref.) | 1 (Ref.) | 1(Ref.) |
|  |  | **Former** | 134,512 | 460 | 0.33 | 1.027 (0.929, 1.137) | 0.745 (0.673, 0.825) | 0.790 (0.714, 0.875) |
|  |  | **Active** | 447,881 | 1,811 | 0.39 | 1.221 (1.146, 1.300) | 0.962 (0.903, 1.026) | 0.983 (0.922, 1.048) |
|  | **40-64** | **None** | 1,354,507 | 18,286 | 1.32 | 1 (Ref.) | 1 (Ref.) | 1 (Ref.) |
|  |  | **Former** | 332,068 | 5,013 | 1.49 | 1.132 (1.097,1.168) | 0.968 (0.936, 1.001) | 0.959 (0.927, 0.992) |
|  |  | **Active** | 486,748 | 9,573 | 1.96 | 1.498 (1.462,1.536) | 1.504 (1.464, 1.546) | 1.448 (1.408, 1.489) |
|  | **≥ 65** | **None** | 370,400 | 31,065 | 8.97 | 1 (Ref.) | 1 (Ref.) | 1(Ref.) |
|  |  | **Former** | 73,270 | 6,774 | 10.37 | 1.181 (1.150, 1.212) | 1.062 (1.032, 1.093) | 1.083 (1.052,1.115) |
|  |  | **Active** | 64,638 | 7,023 | 12.82 | 1.492 (1.454,1.531) | 1.438 (1.398, 1.479) | 1.446 (1.406,1.488) |
|  | *p* for interaction | |  |  |  | <.0001 | <.0001 | <.0001 |
| **Lowest quartile of household income** | **No** | **None** | 1,929,180 | 42,564 | 2.18 | 1 (Ref.) | 1 (Ref.) | 1 (Ref.) |
|  |  | **Former** | 481,084 | 10,348 | 2.14 | 0.988 (0.967,1.009) | 0.986 (0.962, 1.010) | 1.002 (0.976, 1.025) |
|  |  | **Active** | 870,168 | 14,953 | 1.71 | 0.789 (0.774,0.804) | 1.408 (1.377, 1.439) | 1.392 (1.361, 1.424) |
|  | **Yes** | **None** | 413,661 | 8,843 | 2.12 | 1 (Ref.) | 1 (Ref.) | 1 (Ref.) |
|  |  | **Former** | 58,766 | 1,899 | 3.28 | 1.561 (1.486, 1.641) | 1.033 (0.981, 1.087) | 1.039 (0.987, 1.094) |
|  |  | **Active** | 129,099 | 3,454 | 2.71 | 1.294 (1.244, 1.346) | 1.502 (1.442, 1.565) | 1.472 (1.413, 1.534) |
|  | *p* for interaction | |  |  |  | <.0001 | 0.008 | 0.0296 |
| **BMI** (kg/m^2^) | **< 25** | **None** | 1,638,787 | 31,728 | 1.91 | 1 (Ref.) | 1 (Ref.) | 1 (Ref.) |
|  |  | **Former** | 326,036 | 7,771 | 2.39 | 1.255 (1.224, 1.286) | 1.031(1.003, 1.060) | 1.047 (1.018, 1.076) |
|  |  | **Active** | 651,489 | 13,148 | 2.02 | 1.063 (1.041, 1.085) | 1.501(1.466, 1.536) | 1.470 (1.435, 1.505) |
|  | **≥ 25** | **None** | 704,054 | 19,679 | 2.77 | 1 (Ref.) | 1 (Ref.) | 1 (Ref.) |
|  |  | **Former** | 213,814 | 4,476 | 2.08 | 0.755 (0.731, 0.780) | 0.929 (0.898, 0.962) | 0.940 (0.907, 0.973) |
|  |  | **Active** | 347,778 | 5,259 | 1.50 | 0.545 (0.528, 0.562) | 1.284 (1.242, 1.327) | 1.276 (1.235, 1.319) |
|  | *p* for interaction | |  |  |  | <.0001 | <.0001 | <.0001 |
| **Regular exercise** | **No** | **None** | 1,945,468 | 43,176 | 2.20 | 1 (Ref.) | 1 (Ref.) | 1 (Ref.) |
|  |  | **Former** | 403,253 | 8,996 | 2.23 | 1.020 (0.997, 1.044) | 0.988 (0.963, 1.014) | 0.997(0.972, 1.024) |
|  |  | **Active** | 836,915 | 15,166 | 1.81 | 0.828 (0.813, 0.843) | 1.402 (1.371, 1.433) | 1.393(1.362, 1.425) |
|  | **Yes** | **None** | 397,373 | 8,231 | 2.04 | 1 (Ref.) | 1 (Ref.) | 1 (Ref.) |
|  |  | **Former** | 136,597 | 3,251 | 2.37 | 1.169 (1.123, 1.218) | 1.041 (0.998, 1.085) | 1.036 (0.994,1.081) |
|  |  | **Current** | 162,352 | 3,241 | 1.99 | 0.984 (0.945, 1.025) | 1.494 (1.433, 1.558) | 1.470 (1.409,1.533) |
|  | *p* for interaction | |  |  |  | <.0001 | 0.0064 | 0.0381 |
| **Drinking** | **Non** | **None** | 1,619,562 | 41,578 | 2.55 | 1 (Ref.) | 1 (Ref.) | 1 (Ref.) |
|  |  | **Former** | 161,378 | 5,654 | 3.57 | 1.419 (1.380, 1.459) | 1.113 (1.079, 1.148) | 1.106 (1.073, 1.141) |
|  |  | **Active** | 232,746 | 6,501 | 2.82 | 1.121 (1.092, 1.151) | 1.452 (1.411, 1.495) | 1.432 (1.391, 1.474) |
|  | **Mild to**  **moderate** | **None** | 666,651 | 8,346 | 1.23 | 1 (Ref.) | 1 (Ref.) | 1 (Ref.) |
|  |  | **Former** | 306,828 | 5,081 | 1.64 | 1.337 (1.292,1.385) | 0.922 (0.890,0.955) | 0.914 (0.882, 0.947) |
|  |  | **Active** | 588,302 | 8,351 | 1.40 | 1.152 (1.117,1.187) | 1.411 (1.368, 1.456) | 1.358 (1.316, 1.401) |
|  | **Heavy** | **None** | 56,628 | 1,483 | 2.61 | 1 (Ref.) | 1 (Ref.) | 1 (Ref.) |
|  |  | **Former** | 71,644 | 1,512 | 2.10 | 0.803 (0.748,0.863) | 0.910 (0.847, 0.978) | 0.908 (0.845, 0.975) |
|  |  | **Active** | 178,219 | 3,555 | 1.99 | 0.767 (0.722,0.815) | 1.383 (1.302, 1.470) | 1.345 (1.266, 1.429) |
|  | *p* for interaction | |  |  |  | <.0001 | <.0001 | <.0001 |
| **DM** | **No** | **None** | 2,154,376 | 39,386 | 1.80 | 1 (Ref.) | 1 (Ref.) | 1 (Ref.) |
|  |  | **Former** | 479,552 | 9,162 | 1.90 | 1.059 (1.035, 1.083) | 1.004 (0.978, 1.030) | 1.023 (0.997, 1.050) |
|  |  | **Active** | 912,579 | 14,315 | 1.56 | 0.870 (0.854, 0.887) | 1.440 (1.408, 1.473) | 1.418 (1.386, 1.451) |
|  | **Yes** | **None** | 188,465 | 12,021 | 6.63 | 1 (Ref.) | 1 (Ref.) | 1 (Ref.) |
|  |  | **Former** | 60,298 | 3,085 | 5.33 | 0.809 (0.777,0.841) | 0.933 (0.895, 0.972) | 0.958 (0.919, 0.998) |
|  |  | **Active** | 86,688 | 4,092 | 4.93 | 0.751 (0.724,0.778) | 1.362 (1.312, 1.413) | 1.371 (1.321, 1.423) |
|  | *p* for interaction | |  |  |  | <.0001 | 0.0009 | 0.0101 |
| **HTN** | **No** | **None** | 1,741,233 | 23,615 | 1.33 | 1 (Ref.) | 1 (Ref.) | 1 (Ref.) |
|  |  | **Former** | 370,721 | 5,543 | 1.48 | 1.115 (1.083, 1.148) | 0.978 (0.948, 1.009) | 0.997 (0.966, 1.029) |
|  |  | **Active** | 783,618 | 10,410 | 1.31 | 0.994 (0.971, 1.017) | 1.412 (1.376, 1.449) | 1.371 (1.335, 1.407) |
|  | **Yes** | **None** | 601,608 | 27,792 | 4.70 | 1 (Ref.) | 1 (Ref.) | 1 (Ref.) |
|  |  | **Former** | 169,129 | 6,704 | 4.05 | 0.870 (0.847, 0.893) | 0.999 (0.970, 1.028) | 1.014 (0.985, 1.044) |
|  |  | **Active** | 215,649 | 7,997 | 3.81 | 0.821 (0.800, 0.841) | 1.480 (1.441, 1.522) | 1.451 (1.412, 1.492) |
|  | *p* for interaction | |  |  |  | <.0001 | 0.0218 | 0.0042 |
| **Dyslipidemia** | **No** | **None** | 1,920,601 | 36,294 | 1.86 | 1 (Ref.) | 1 (Ref.) | 1 (Ref.) |
|  |  | **Former** | 436,988 | 9,128 | 2.08 | 1.121 (1.096, 1.147) | 0.986 (0.961, 1.011) | 1.007 (0.981, 1.033) |
|  |  | **Active** | 851,595 | 14,555 | 1.70 | 0.918 (0.901, 0.936) | 1.424 (1.393, 1.456) | 1.406 (1.375, 1.439) |
|  | **Yes** | **None** | 422,240 | 15,113 | 3.57 | 1 (Ref.) | 1(Ref.) | 1(Ref.) |
|  |  | **Former** | 102,862 | 3,119 | 3.05 | 0.860 (0.827, 0.894) | 1.001 (0.960, 1.041) | 1.004 (0.964, 1.046) |
|  |  | **Active** | 147,672 | 3,852 | 2.62 | 0.741 (0.715, 0.768) | 1.432 (1.379, 1.486) | 1.409 (1.357, 1.463) |
|  | *p* for interaction | |  |  |  | <.0001 | 0.8212 | 0.9879 |
| **CKD** | **No** | **None** | 2,164,555 | 41,946 | 1.91 | 1 (Ref.) | 1 (Ref.) | 1 (Ref.) |
|  |  | **Former** | 498,896 | 10,330 | 2.06 | 1.085 (1.062, 1.108) | 0.983 (0.960, 1.008) | 1.001 (0.976, 1.026) |
|  |  | **Active** | 949,595 | 16,553 | 1.73 | 0.915 (0.899, 0.932) | 1.440 (1.410, 1.471) | 1.416 (1.385, 1.447) |
|  | **Yes** | **None** | 178,286 | 9,461 | 5.46 | 1 (Ref.) | 1 (Ref.) | 1 (Ref.) |
|  |  | **Former** | 40,954 | 1,917 | 4.85 | 0.890 (0.848, 0.935) | 1.017 (0.966, 1.070) | 1.039 (0.988, 1.093) |
|  |  | **Active** | 49,672 | 1,854 | 3.82 | 0.699 (0.665, 0.735) | 1.331 (1.265, 1.401) | 1.341 (1.274, 1.411) |
|  | *p* for interaction | |  |  |  | <.0001 | 0.0031 | 0.0282 |

Model 1: Non-adjusted, Model 2: Adjusted with age and sex, Model 3: Adjusted with age, sex, income status of the lowest quartile, five categories of body-mass index, regular exercise, alcohol consumption, diabetes mellitus, hypertension, dyslipidemia, and chronic kidney disease. ^a^ Per 1,000 person-years.

Aberrations: CKD, chronic kidney disease; CI, confidence interval; DM, diabetes mellitus; HR, hazard ratio; HTN, hypertension; IR, incidence rate; Ref, reference.

**Supplementary Fig. 1**. Impact of total smoking amount on incidence of sepsis by baseline characteristics

(A) Sex


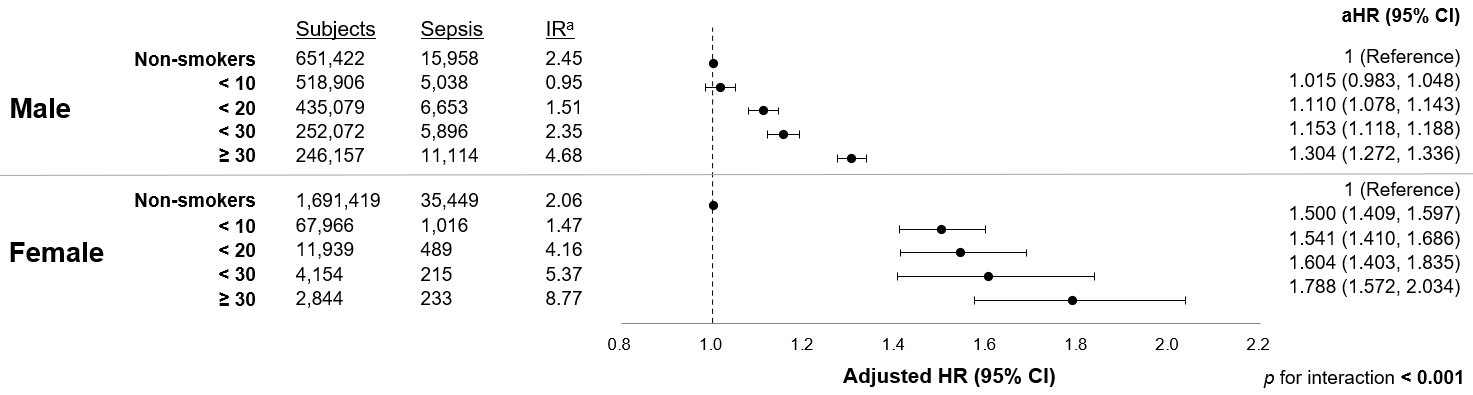


(B) Age group


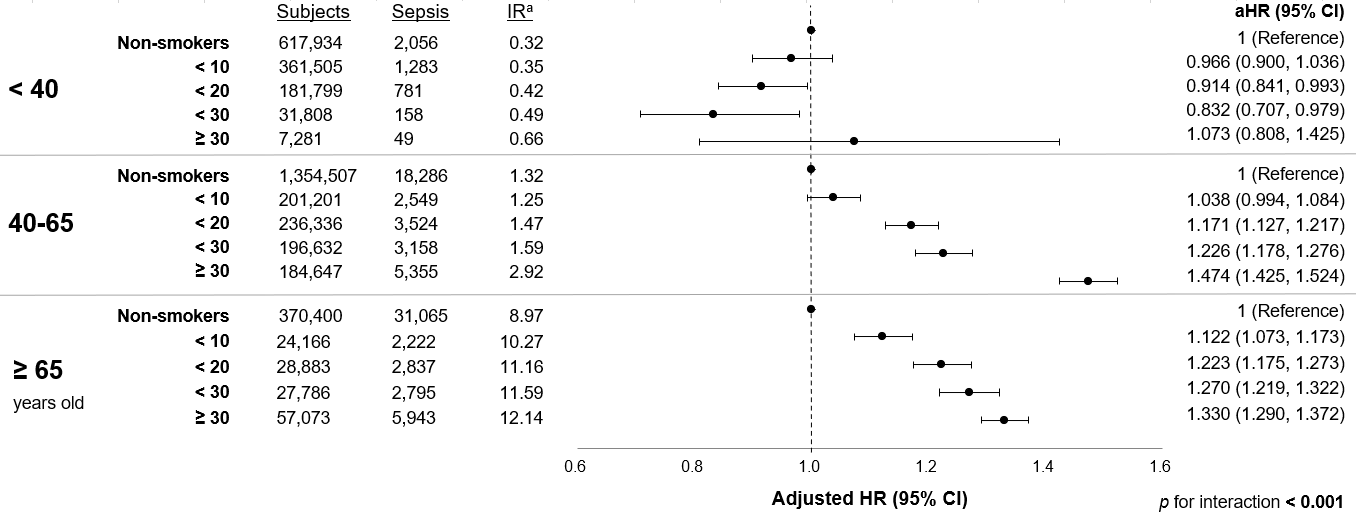


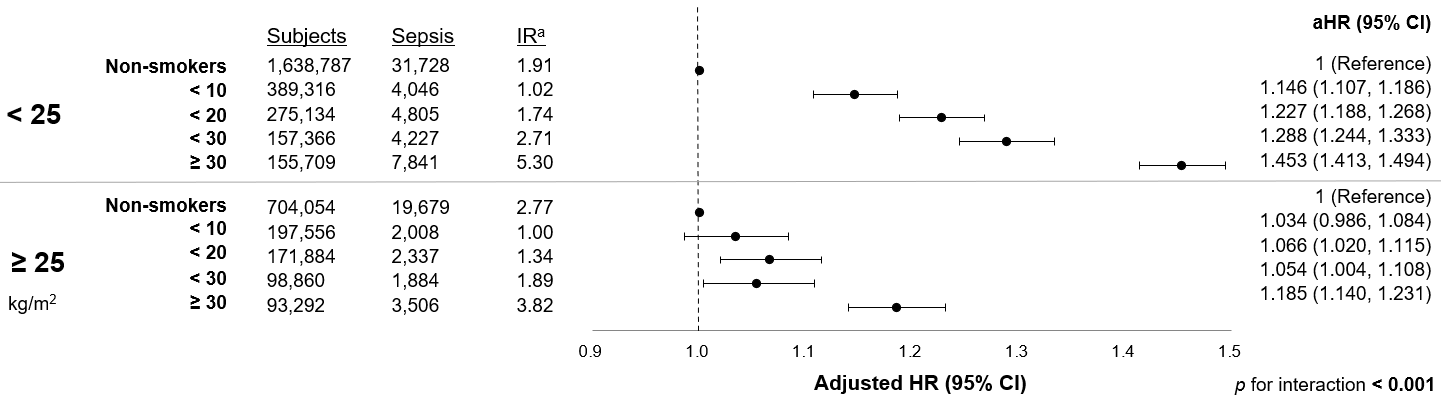
(C) BMI

(D) Lowest income status
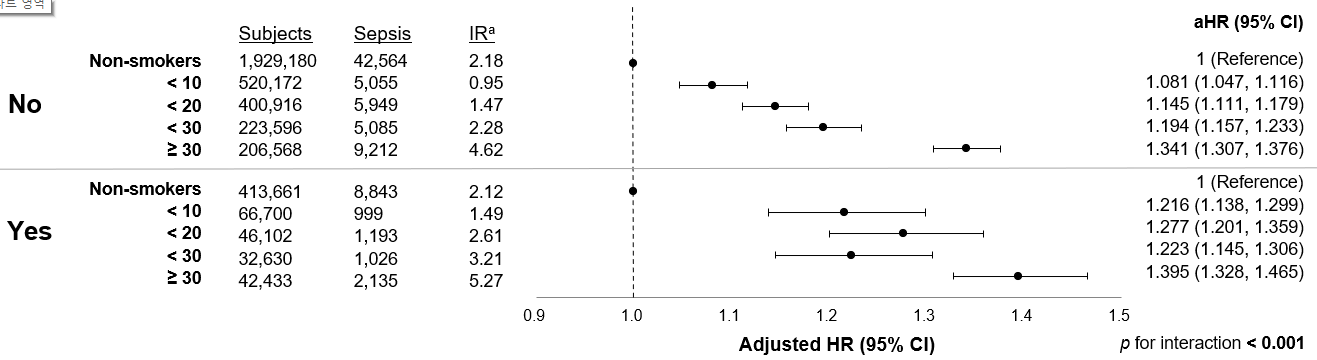


**Supplementary Fig. 2**. Impact of total smoking amount on incidence of sepsis by lifestyle behaviors

(A) Regular exercise


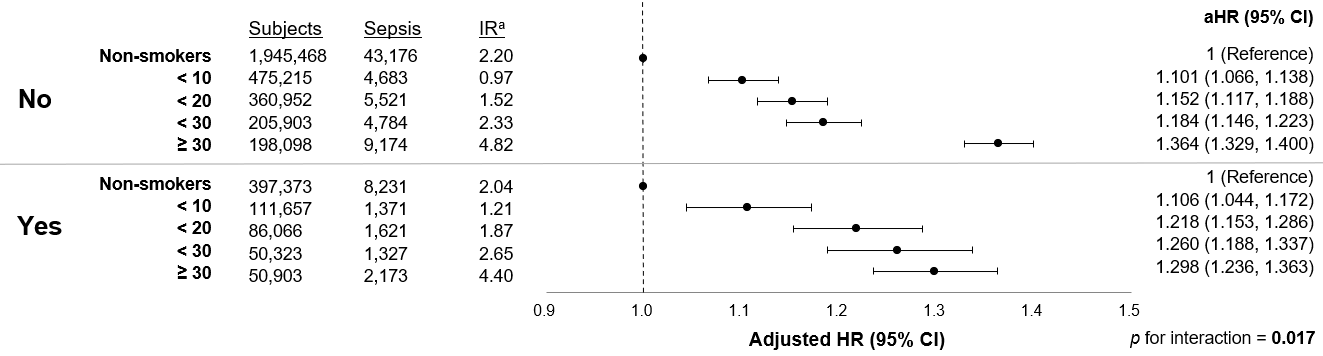


(B) Drinking


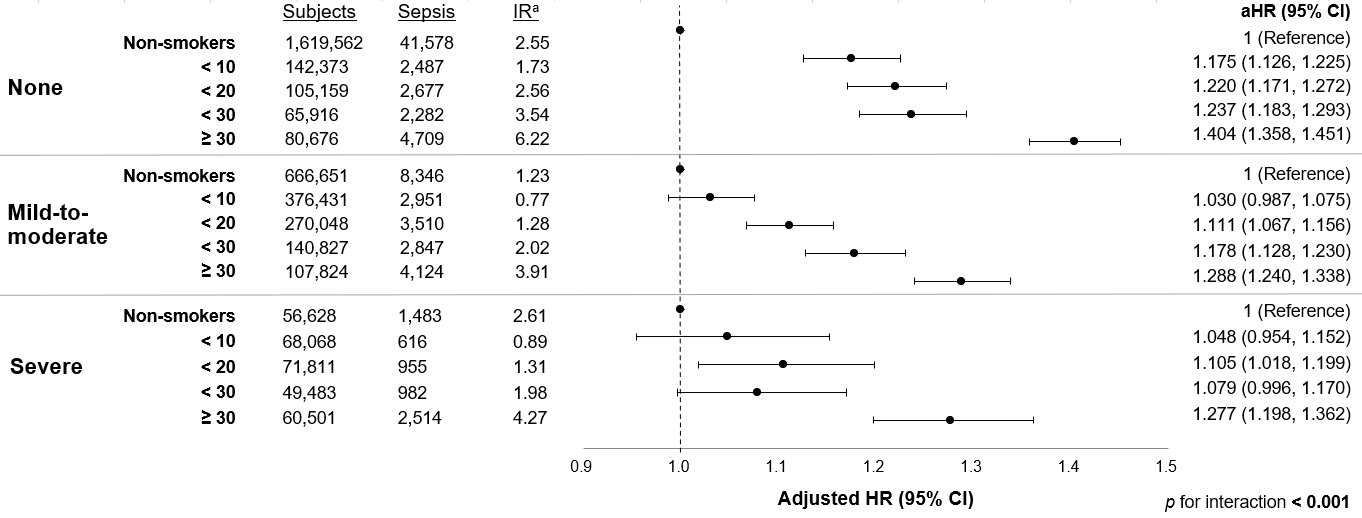


**Supplementary Fig. 3**. Impact of total smoking amount on incidence of sepsis by medical comorbidities


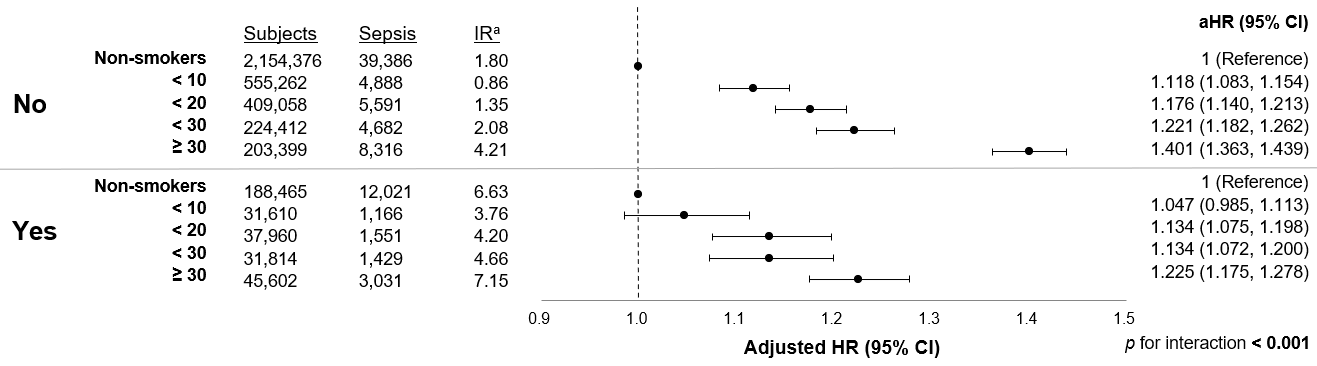

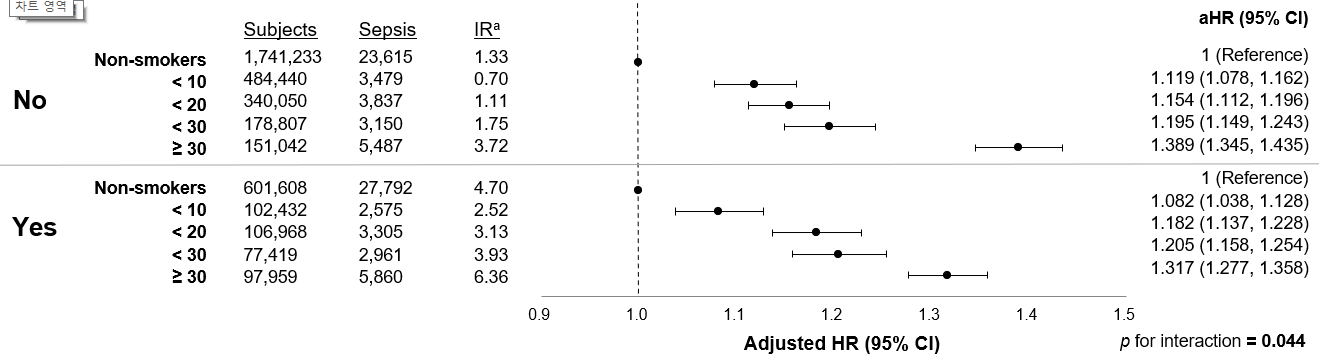
(A) Diabetes mellitus

(B) Hypertension


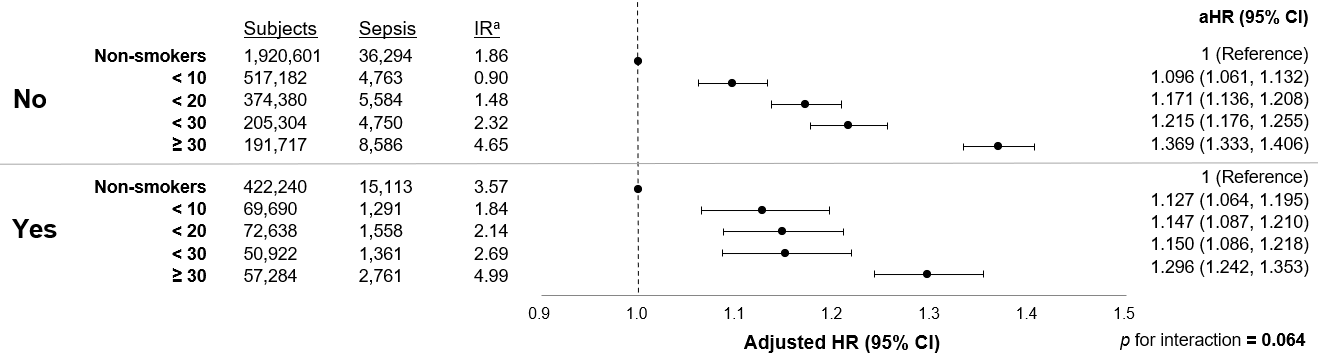
(C) Dyslipidemia


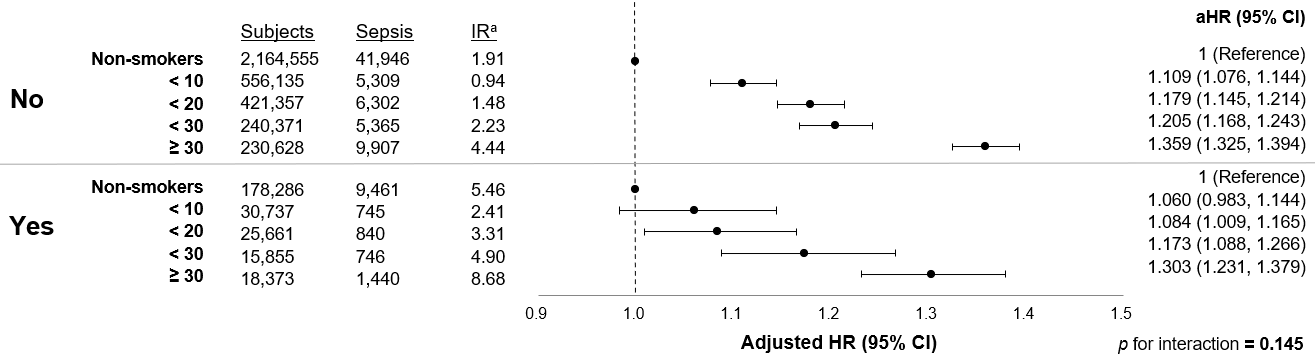
(D) Chronic kidney disease
